# MAP-Derived Shock Index for Point-of-Care Physiological Risk Stratification After CT-Confirmed Cervical Spine Fracture: Development and Internal Validation of a Prognostic Model for In-Hospital Mortality

**DOI:** 10.3390/diagnostics16142272

**Published:** 2026-07-21

**Authors:** Mustafa Safa Pepele, Melike Karataş Ayhan, Serdar Derya, Mahmut Murat, Şükrü Gürbüz, Neslihan Yücel

**Affiliations:** Department of Emergency Medicine, Faculty of Medicine, İnönü University, Malatya 44280, Türkiye; melike.karatas@inonu.edu.tr (M.K.A.); serdar.derya@inonu.edu.tr (S.D.); mahmut.murat@inonu.edu.tr (M.M.); sukru.gurbuz@inonu.edu.tr (Ş.G.); neslihan.yucel@inonu.edu.tr (N.Y.)

**Keywords:** cervical spine fracture, computed tomography, MAP-derived shock index, physiological risk stratification, prognostic model, trauma mortality, emergency department, clinical decision support

## Abstract

**Background/Objectives**: Early mortality risk stratification after CT-confirmed cervical spine fractures remains clinically challenging in patients with major trauma. Imaging establishes a structural diagnosis, but it does not fully capture early physiological deterioration. This study aimed to develop and internally validate a prognostic model for in-hospital mortality and evaluate whether a MAP-derived Shock Index provides incremental prognostic information beyond routinely available clinical variables and the Injury Severity Score (ISS). **Methods**: This retrospective single-center cohort study included 131 adults with CT-confirmed cervical spine fractures and ISS ≥ 15 who were admitted to a tertiary trauma center between 2013 and 2023. The MAP-derived Shock Index was calculated as heart rate divided by estimated systolic blood pressure, where estimated systolic blood pressure was defined a priori as 1.5 × MAP. Four prespecified logistic regression models were evaluated: a base clinical model, base plus MAP-derived Shock Index, base plus ISS, and base plus MAP-derived Shock Index plus ISS. Discrimination, Brier score, calibration, bootstrap internal validation, and decision curve analyses were performed. **Results**: The in-hospital mortality rate was 22.9% (30/131). The base plus MAP-derived Shock Index model achieved an apparent AUC of 0.93 and an optimism-corrected AUC of 0.91. The MAP-derived Shock Index provided incremental prognostic information beyond the ISS; as an individual predictor, its AUC was 0.82 compared with 0.74 for the ISS. With each 0.1-unit increase, the MAP-derived Shock Index was associated with higher odds of in-hospital mortality. Decision curve analysis suggested a higher apparent net benefit across clinically relevant thresholds. **Conclusions**: In this internally validated retrospective cohort, the MAP-derived Shock Index complemented clinical and ISS-based assessments for early in-hospital mortality risk stratification after CT-confirmed cervical spine fracture. The index is not the conventional SBP-based Shock Index, and the model requires external multicenter validation before clinical use.

## 1. Introduction

Cervical spine fractures are clinically important traumatic injuries associated with substantial morbidity, intensive care, and in-hospital mortality. Computed tomography (CT) confirms the structural diagnosis and guides subsequent anatomical assessment; however, early mortality risk stratification in the emergency department (ED) remains challenging. In this early phase, clinicians must rapidly identify patients requiring intensified monitoring, airway or hemodynamic support, intensive care unit prioritization, or transfer to a higher-level trauma center.

Existing cervical spine trauma frameworks, including the Subaxial Injury Classification System and AO Spine classification, are essential for describing fracture morphology, spinal stability, and surgical decision-making [1,2,3]. However, these systems are not primarily designed to quantify immediate systemic and physiological deterioration. Similarly, the Injury Severity Score (ISS) summarizes the global anatomical injury burden but may not fully reflect early hemodynamic compromise or neurogenic/hemorrhagic shock physiology [4,5].

Vital-sign-based indices have been evaluated in general trauma and emergency populations as pragmatic tools for early risk assessment [6,7,8,9,10,11]. The conventional Shock Index is calculated as the heart rate divided by the systolic blood pressure [6,7,8]. In the present study, direct systolic blood pressure was not consistently available as a separate structured variable; therefore, we evaluated a prespecified MAP-derived Shock Index calculated from heart rate and MAP-derived estimated systolic pressure. This distinction is central to the interpretation and generalizability of these findings. Neurological classification, spinal cord injury pathophysiology, cervical injury severity frameworks, and timing of decompression also remain important components of prognosis and management in cervical spine trauma [12,13,14,15,16]. Other trauma scoring systems incorporating physiological variables have also been proposed for early mortality prediction [17].

Accordingly, this study was designed to determine whether the MAP-derived Shock Index adds prognostic information beyond routinely available clinical variables and ISS for predicting in-hospital mortality after CT-confirmed cervical spine fractures in patients with major trauma. We hypothesized that a physiology-augmented model would improve early mortality risk stratification while recognizing that such a model should complement rather than replace anatomical and neurological assessments.

## 2. Materials and Methods

### 2.1. Study Design and Setting

This retrospective, single-center cohort study was conducted in the emergency department of a tertiary academic trauma center in Türkiye. The center functions as a regional referral hospital for adult trauma, spinal trauma, and neurotrauma patients. The study period was from January 2013 to December 2023.

The study was conducted in accordance with the principles of the Declaration of Helsinki and approved by the İnönü University Scientific Research and Publication Ethics Committee, Health Sciences Scientific Research Ethics Committee (Decision No. 2025/8367; approval date: 16 September 2025). Because of the retrospective design and use of anonymized routine clinical data, the requirement for individual informed consent was waived by the Ethics Committee.

This study was reported in accordance with the Transparent Reporting of a Multivariable Prediction Model for Individual Prognosis or Diagnosis (TRIPOD) statement for prognostic prediction model studies [18]. The primary objective was to evaluate whether early physiological information available at emergency department presentation improved in-hospital mortality prediction after CT-confirmed cervical spine fractures in patients with major trauma.

### 2.2. Eligibility Criteria and Study Cohort

All adult patients aged 18 years or older who presented to the emergency department during the study period and had a computed tomography-confirmed cervical spine fracture were screened for eligibility criteria. To focus on clinically relevant major trauma, inclusion was restricted to patients with an Injury Severity Score (ISS) ≥ 15.

Patients were excluded if they had penetrating neck trauma, thoracic or lumbar spinal fractures without cervical spine involvement, cardiac arrest or death before completion of the initial emergency department assessment, missing physiological data required for MAP-derived Shock Index calculation, or incomplete hospital outcome documentation. After applying these criteria, 131 patients were included in the study.

The patient selection process is illustrated in Supplementary Figure S1.

### 2.3. Data Sources and Variables

Data were extracted from the hospital electronic medical record system, emergency department records, trauma registry files, and radiology database. Cervical spine fracture diagnosis was confirmed by reviewing computed tomography reports in the institutional radiology system.

The following variables were recorded at the time of presentation to the emergency department: age, sex, mechanism of injury, fracture level, heart rate, mean arterial pressure (MAP), respiratory rate, peripheral oxygen saturation, temperature, Glasgow Coma Scale (GCS) score, emergency department intubation status, comorbidity status, ISS, arterial lactate, and base deficit. The available retrospective dataset did not contain reliably documented American Spinal Injury Association (ASIA) Impairment Scale scores or complete spinal cord injury level data; therefore, these variables could not be included in the primary model.

Comorbidity was defined as the presence of at least one documented chronic systemic disease in the medical history. Emergency department intubation was recorded as a dichotomous variable. The primary outcome was all-cause in-hospital mortality during index admission.

### 2.4. Source of Blood Pressure Data and Definition of the MAP-Derived Shock Index

The conventional Shock Index is defined as the heart rate divided by the systolic blood pressure and has been widely evaluated as an early physiological marker in emergency and trauma populations [6,7,8]. In the institutional source dataset used for the present analysis, direct systolic blood pressure was not consistently available as a separate structured variable, whereas the early arterial pressure field exported from the electronic record was labelled MAP. To preserve a prespecified pressure-adjusted heart rate index while maintaining transparency, the estimated systolic blood pressure was derived as 1.5 × MAP, and the MAP-derived Shock Index was calculated as heart rate divided by this estimated systolic pressure.

The MAP-derived Shock Index was calculated as follows:

MAP-derived Shock Index = HR/(1.5 × MAP)

where HR denotes heart rate and MAP denotes mean arterial pressure.

Therefore, this variable is not the conventional SBP-based Shock Index and should be interpreted as a MAP-derived approximation. This definition was consistently applied to all patients. The measurement and interpretability implications of using this MAP-derived index are addressed in detail in the Discussion and Limitations sections of this paper.

### 2.5. Model Development

Four prespecified logistic regression models were developed to predict in-hospital mortality.

1. Base model: age, Glasgow Coma Scale score, comorbidity, and emergency department intubation.
2. Base + MAP-derived Shock Index model: base model plus MAP-derived Shock Index;
3. Base + ISS model: base model plus Injury Severity Score.
4. Base + MAP-derived Shock Index + ISS model: base model plus both MAP-derived Shock Index and Injury Severity Score.

The base model variables were selected a priori according to clinical relevance, availability during the early emergency department assessment, and prior trauma prognostication literature [6,7,8,9,10,11,17]. Continuous variables were retained on their original scales and not categorized. Restricted cubic spline terms were explored for continuous predictors, and linear terms were retained when the non-linear terms did not improve the model fit or calibration. Detailed cervical morphology, ASIA Impairment Scale grade, and spinal cord injury level were not included because they were not reliably available in the structured retrospective records.

Because the number of events was limited, the model complexity was intentionally restricted. Penalized logistic regression using ridge regularization was performed as a sensitivity analysis to assess the robustness of primary maximum-likelihood estimates.

### 2.6. Handling of Missing Data

The completeness of the dataset exceeded 95% for the primary candidate predictors and outcomes. Patients with missing essential variables required for the primary models or missing in-hospital outcome status were excluded from the final analyses. The variable-level missingness is provided in Supplementary Table S4.

Because the primary analysis was based on complete cases and the amount of missing data was low, multiple imputation was not performed. Sensitivity analyses were used to assess whether the main findings were robust to the model specifications and penalization.

### 2.7. Statistical Analysis

Continuous variables are presented as the median with interquartile range or mean with standard deviation, depending on the distribution. Categorical variables are presented as counts and percentages. Baseline characteristics were summarized descriptively, and no formal hypothesis testing was performed for survivor-versus-non-survivor comparisons.

Model discrimination was assessed using the area under the receiver operating characteristic curve. The overall prediction error was assessed using the Brier score. Calibration was evaluated using calibration-in-the-large, calibration slope, and calibration plots comparing observed and predicted mortality risks across risk strata.

Internal validation was performed using bootstrap resampling with 1000 repetitions. Optimism in the model performance was estimated and subtracted from the apparent performance to obtain optimism-corrected estimates. A uniform shrinkage factor based on the optimism-corrected calibration slope was then applied to the final model coefficients, and the intercept was recalibrated to the observed event rates.

Model comparisons focused on the incremental prognostic value of the MAP-derived Shock Index over the base model and ISS-based prediction. Reclassification was assessed using continuous net reclassification improvement and integrated discrimination improvement. For clinical interpretability, the association between the MAP-derived Shock Index and in-hospital mortality was additionally reported per 0.1-unit increase, rather than only per 1-unit increase.

### 2.8. Decision-Curve Analysis Methodology

The clinical utility was evaluated using decision-curve analysis across threshold probabilities from 5% to 50%, following the established net-benefit methodology [19,20]. The net benefit was calculated from the apparent predicted probabilities of each fitted model without smoothing or interpolation. The treat-all and treat-none strategies were included as reference strategies. Because the decision curves are based on apparent predictions from an internally validated cohort, they should be interpreted as exploratory and require external validation in future studies.

Threshold probabilities between 10% and 25% were considered clinically relevant for early emergency department decision-making, including intensive monitoring, early intensive care unit prioritization, and escalation of resuscitation care.

### 2.9. Software and AI-Assisted Manuscript Preparation

All analyses were performed using Python 3.11. Statistical modeling and performance assessment were conducted using StatsModels version 0.14.4, NumPy version 2.0.2, Pandas version 2.2.2, Matplotlib version 3.9.2, and Scikit-learn version 1.5.1. The random seeds were fixed to ensure reproducibility. The analysis code and an Excel-based bedside risk calculator derived from the final shrinkage-adjusted model are provided as Supplementary Material.

No chemicals, reagents, commercial cell lines, or study-specific experimental materials were used in this retrospective clinical study. The analysis was based on routinely collected clinical records, trauma registry data, and radiology reports.

A generative AI tool, OpenAI ChatGPT (GPT-5.5 Thinking, OpenAI, San Francisco, CA, USA; accessed July 2026), was used only as auxiliary editorial and formatting support during manuscript revision, including language refinement, organization, and preparation of the supplementary presentation materials. The tool was not used for data collection, clinical decision-making, study design, statistical analysis, or independent interpretation of the results. All outputs were reviewed, verified, and edited by the authors.

## 3. Results

### 3.1. Study Population

A total of 131 adult patients with computed tomography-confirmed cervical spine fractures and major trauma severity, defined as an Injury Severity Score (ISS) ≥ 15, were included in the final analysis. The in-hospital mortality rate was 22.9% (30/131). The median ISS was 34 (interquartile range [IQR], 26–43), confirming that the cohort represented a high severity trauma population.

The median age was 56 years (IQR, 38–69 years), and 98 patients (74.8%) were male. The most common mechanisms of injury were motor vehicle-related trauma (71.0%) and falls (26.0%). Upper cervical fractures were present in 52 patients (39.7%), whereas subaxial or other cervical fractures were observed in 79 patients (60.3%). Emergency department intubation was performed in 38 patients (29.0%), and 77 patients (58.8%) had at least one comorbidity.

Non-survivors were older, had higher ISS values, more frequent comorbidities, more frequent emergency department intubation, and higher MAP-derived Shock Index values than survivors. Baseline demographic, injury, and emergency department characteristics are presented in Table 1.

### 3.2. Model Performance

The base clinical model, which included age, Glasgow Coma Scale score, comorbidity, and emergency department intubation, showed good discrimination for in-hospital mortality, with an apparent area under the receiver operating characteristic curve (AUC) of 0.86 and an optimism-corrected AUC of 0.84. The apparent Brier score was 0.12.

Adding the MAP-derived Shock Index to the base model improved the apparent model performance. The base + MAP-derived Shock Index model achieved an apparent AUC of 0.93 and an optimism-corrected AUC of 0.91 after bootstrap internal validation. This model also had a lower Brier score than the base model (0.09 vs. 0.12, respectively). These findings indicate incremental prognostic information from early physiology in this cohort, rather than the replacement of anatomical or neurological assessment.

The base + ISS model showed only a modest improvement over the base model, with an apparent AUC of 0.87 and an optimism-corrected AUC of 0.84. The combined base + MAP-derived Shock Index + ISS model did not meaningfully improve discrimination beyond the base + MAP-derived Shock Index model (apparent AUC, 0.93; optimism-corrected AUC, 0.90).

**Figure 1:**
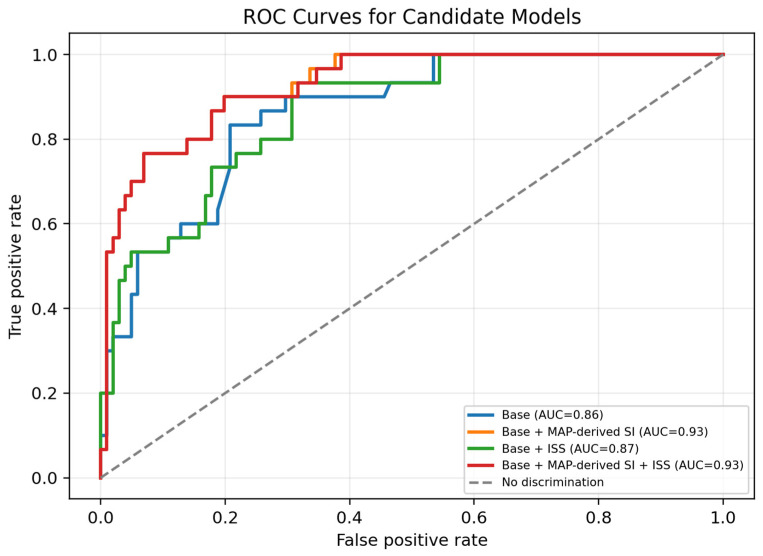
ROC Curves for the Candidate Models. Caption: Receiver operating characteristic (ROC) curves comparing the apparent discrimination of candidate models for in-hospital mortality prediction. The Base + MAP-derived Shock Index (SI) model achieved an apparent AUC of 0.93, compared with 0.86 for the Base model and 0.87 for the Base + ISS models. The dashed diagonal line represents the line of no discrimination. Abbreviations: AUC = area under the ROC curve; SI = MAP-derived Shock Index; ISS = Injury Severity Score; ED = emergency department.

The receiver operating characteristic curves of the candidate models are shown in Figure 1. The apparent and optimism-corrected model performance metrics are summarized in Table 2.

### 3.3. Comparison Between MAP-Derived Shock Index and Injury Severity Score

When evaluated as individual predictors, the MAP-derived Shock Index showed higher apparent discrimination for in-hospital mortality than the ISS alone, with AUC values of 0.82 and 0.74, respectively. When added to the base clinical model, the MAP-derived Shock Index produced a larger improvement in discrimination than the ISS in this retrospective cohort.

The MAP-derived Shock Index was strongly associated with in-hospital mortality. Because the odds ratio per 1-unit increase is difficult to interpret clinically, the effect was additionally expressed per 0.1-unit increase (OR 1.72, 95% CI 1.29–2.30). The apparent difference in AUC between the base + MAP-derived Shock Index model and the base + ISS model was approximately 0.06. Reclassification analyses were consistent with this finding, although they should be interpreted cautiously, given the modest sample size and internal validation only.

The correlation between the MAP-derived Shock Index and ISS was moderate, suggesting that these variables capture related but non-identical dimensions of trauma severity. Whereas the ISS reflects the anatomical injury burden, the MAP-derived Shock Index reflects early physiological derangement at emergency department presentation.

### 3.4. Calibration and Internal Validation

Bootstrap internal validation demonstrated that the base + MAP-derived Shock Index model retained a strong performance after optimism correction. The optimism-corrected AUC was 0.91, supporting limited optimism in the primary physiology-augmented model.

A uniform shrinkage factor derived from the optimism-corrected calibration slope was applied to the final model coefficients, and the intercept was recalibrated to the observed in-hospital mortality rates. Calibration plots showed acceptable agreement between the predicted and observed mortality risk across most risk strata, with wider uncertainty at the highest predicted-risk range because of the limited number of patients in these strata.

The final shrinkage-adjusted model coefficients are presented in Supplementary Table S1.

### 3.5. Decision-Curve Analysis

Decision curve analysis was performed using the standard net benefit formula and apparent model predictions from each fitted model. The base + MAP-derived Shock Index model showed a higher apparent net benefit than the base model and the base + ISS model across a broad range of threshold probabilities. The advantage was most evident between 10% and 25%, a range that may be relevant for early emergency department decisions, such as intensive monitoring, intensive care unit prioritization, and escalation of resuscitative care.

**Figure 2:**
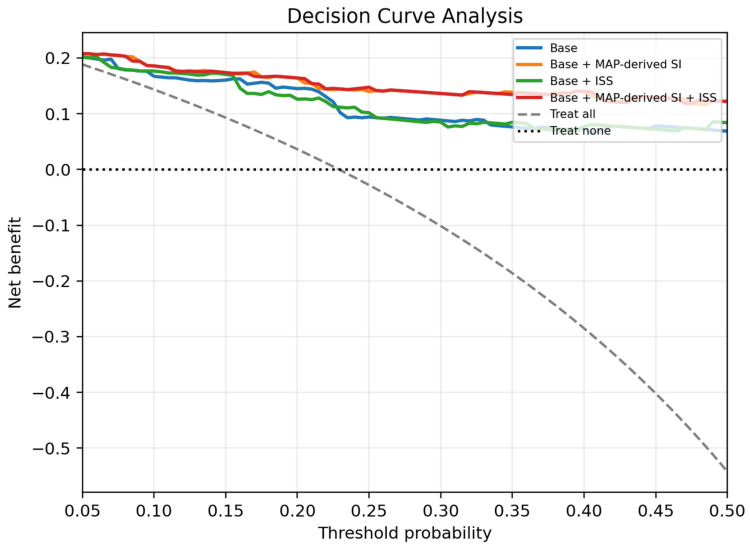
Decision Curve Analysis (DCA). Decision curve analysis comparing the apparent net benefit across threshold probabilities (5–50%) for the Base, Base + MAP-derived SI, Base + ISS, and Base + MAP-derived SI + ISS models. The net benefit was calculated using the standard decision-curve methodology from the apparent predicted probabilities; no smoothing or interpolation was applied. The treat-all and treat-none reference strategies are shown. The partial overlap between the Base + MAP-derived SI and Base + MAP-derived SI + ISS curves reflects their similar apparent net benefit and does not affect the scientific interpretation of the figure. The Base + MAP-derived SI and Base + MAP-derived SI + ISS curves were similar, indicating limited incremental decision-curve value from adding ISS to the MAP-derived SI model. Abbreviations: DCA, decision-curve analysis; SI, MAP-derived Shock Index; ISS, Injury Severity Score; ICU, intensive care unit.

At a 15% risk threshold, the base + MAP-derived Shock Index model was associated with fewer unnecessary high-risk classifications than the base model while preserving the identification of patients at elevated mortality risk. Because these curves are based on apparent predictions from the development cohort, the decision curve findings should be considered exploratory. The decision curve analysis is shown in Figure 2.

### 3.6. Sensitivity and Exploratory Analyses

Sensitivity analyses supported the robustness of the primary results. Penalized logistic regression using ridge regularization yielded model rankings consistent with primary logistic regression models. Exploratory models incorporating available cervical-level information and cervical dislocation did not materially change the overall interpretation, although these variables were incomplete proxies and not substitutes for detailed cervical morphology or ASIA Impairment Scale grading. Laboratory-augmented exploratory models incorporating lactate or base deficit provided only modest additional improvement and did not change the overall ranking of candidate models.

Influence diagnostics did not identify any observation that materially altered the main model coefficients, discrimination, or calibration. These analyses support the stability of the physiology-augmented model, despite the modest number of outcome events.

### 3.7. Implementation Resource

An Excel-based bedside risk calculator was derived from the final shrinkage-adjusted base + MAP-derived Shock Index model using the shrinkage-adjusted coefficients and recalibrated intercept. The calculator allows individualized estimation of in-hospital mortality risk using variables available during the early emergency department assessment: age, GCS score, comorbidity, emergency department intubation status, and MAP-derived Shock Index. This tool is clearly labelled as a research and external validation resource and should not be used as a stand-alone clinically validated decision rule.

## 4. Discussion

### 4.1. Principal Findings

In this retrospective cohort of adults with CT-confirmed cervical spine fractures and major trauma severity, a physiology-augmented model incorporating the MAP-derived Shock Index improved the prediction of in-hospital mortality beyond routinely available clinical variables. The base model already showed good discrimination, and the addition of the MAP-derived Shock Index improved the apparent and optimism-corrected performance. These findings suggest that early physiological derangement contributes prognostic information during the post-diagnostic phase of cervical spine trauma care.

The incremental value of adding ISS to the MAP-derived Shock Index model was small. This should not be interpreted as evidence that anatomical injury assessment is less important than physiological assessment. The ISS is a global anatomical severity measure and is not equivalent to detailed cervical fracture morphology, SLICS or AO Spine classification, spinal cord injury level, or operative decision-making variables. The present findings support complementarity: early physiology may add mortality risk information beyond global anatomical severity in this cohort.

Importantly, the model was developed using variables available during the early emergency department assessment and was only internally validated. Therefore, it should be interpreted as a hypothesis-generating prognostic tool requiring external multicenter validation, not as a model ready for routine clinical implementation.

### 4.2. Interpretation in the Context of Previous Literature

Risk assessment of cervical spine trauma has traditionally emphasized anatomical classification systems, fracture morphology, spinal stability, and neurological injury severity. Systems such as the Subaxial Injury Classification System and AO Spine classification are essential for describing structural injuries and guiding definitive management [1,2,3]. However, these systems are not primarily designed to detect early systemic physiological deterioration.

The conventional Shock Index reflects the relationship between heart rate and systolic blood pressure and has been widely studied in emergency and trauma populations [6,7,8,9,10,11,17]. The present study differs from the literature because the central predictor was a MAP-derived approximation rather than the conventional SBP-based Shock Index. Consequently, the results should not be directly extrapolated to the conventionally calculated Shock Index without validation in datasets containing complete systolic blood pressure measurements.

Cervical spine trauma has a distinct physiological profile. Neurogenic shock, hemorrhagic shock, respiratory compromise, and associated multisystem injuries may coexist, making early bedside assessment complex [12,13]. In this setting, an anatomical score alone may underestimate the prognostic importance of evolving physiological instability. Our findings suggest that the MAP-derived Shock Index may capture a clinically relevant component of early risk that complements the structural injury assessment.

### 4.3. Diagnostic and Prognostic Implications

From a Diagnostics perspective, this study should be interpreted within the broader diagnostic pathway of cervical spine trauma. Computed tomography establishes the structural diagnosis of cervical spine fractures; however, imaging alone does not fully determine the early mortality risk. After diagnosis confirmation, clinicians must rapidly identify patients requiring intensified monitoring, early airway or hemodynamic support, intensive care unit prioritization, or transfer to a higher-level trauma center.

The MAP-derived Shock Index may serve as a simple adjunctive prognostic marker in the post-diagnostic phase. Its potential advantage is that it can be calculated from routinely collected early vital sign data and incorporated into electronic triage systems or research calculators. Decision curve analysis suggests a possible apparent net benefit across clinically relevant risk thresholds; however, these findings were based on apparent predictions and require external validation before clinical application [19,20].

Nevertheless, these findings should not be interpreted as supporting the MAP-derived Shock Index as a stand-alone decision rule. Rather, it should be considered as one component of a broader clinical assessment that includes neurological status, CT findings, cervical fracture morphology, spinal cord injury characteristics, comorbidities, airway requirements, injury mechanism, and institutional trauma resources.

### 4.4. Strengths

This study had several strengths. First, it focused on a clinically high-risk population: patients with CT-confirmed cervical spine fractures and ISS ≥ 15. This approach reduced the heterogeneity related to minor trauma and allowed the analysis to target patients in whom early mortality prediction was clinically meaningful.

Second, this study evaluated discrimination, calibration, Brier score, optimism correction, and decision curve analysis. This is important because a prognostic model with good discrimination may be poorly calibrated or clinically unhelpful. Third, the model was intentionally restricted to variables available during the early emergency department assessment, increasing its potential clinical applicability.

Fourth, internal validation with bootstrap resampling and shrinkage adjustment was used to reduce optimism and to address the risk of overfitting. Finally, the inclusion of an Excel-based calculator may facilitate transparent external validation and future prospective testing, provided that it is not used as a stand-alone clinical decision rule.

### 4.5. Limitations

This study had some important limitations. First, it was a single-center retrospective study, which limits its generalizability. Local trauma workflows, referral patterns, imaging protocols, documentation practices, and intensive care availability may influence both the mortality risk and model performance.

Second, the number of outcome events was small. The cohort included 131 patients, of whom 30 died in the hospital. Although the model complexity was restricted and internal validation with shrinkage was applied, the risk of overfitting could not be fully eliminated, particularly because the final model included several predictors. Therefore, the model should be considered internally validated and hypothesis-generating, and not ready for routine clinical implementation.

Third, the main predictor was not the conventional SBP-derived Shock Index. Direct systolic blood pressure values were not consistently available as separate structured variables in the exported dataset. Therefore, the MAP-derived Shock Index was calculated using the estimated systolic pressure derived from the MAP (1.5 × MAP). Although this definition was prespecified and applied consistently, it may have introduced measurement errors and limited comparability with studies using conventionally measured systolic blood pressure. Future studies should directly compare the conventional Shock Index, MAP-derived Shock Index, and heart rate/MAP ratios in the same patients.

Fourth, ASIA scores, detailed spinal cord injury classifications, fracture morphology, radiological instability, and operative management variables were not systematically documented across the entire study period and could not be reliably incorporated into the final models. Consequently, we were unable to adjust for neurological injury severity or to separately analyze patients with spinal cord injury and those with isolated bony or ligamentous injuries. Therefore, residual confounding related to neurological presentation cannot be excluded. The MAP-derived Shock Index should be interpreted only as an adjunctive marker of early physiological derangement and not as a replacement for neurological examination, ASIA grading, detailed imaging-based classification, or surgical assessment.

Fifth, the outcome was limited to in-hospital mortality only. The model was not designed to predict neurological recovery, functional outcomes, long-term survival, surgical benefits, or rehabilitation needs. In addition, the decision curve analysis used apparent predicted probabilities from the development cohort; therefore, its clinical utility should be confirmed in external validation cohorts before implementation.

### 4.6. Future Directions

Future studies should externally validate this physiology-augmented model in larger, multicenter cohorts with standardized vital sign recordings and complete documentation of systolic blood pressure, mean arterial pressure, and other early hemodynamic variables. Validation should compare the conventionally calculated Shock Index, MAP-derived Shock Index, and heart rate/MAP ratio to determine whether the present pressure-derived approach performs similarly in independent datasets.

Further research should evaluate whether combining physiological variables with detailed CT-based fracture morphology, SLICS or AO Spine classification, ASIA Impairment Scale grade, spinal cord injury level, operative management, and early laboratory markers improves the prognostic accuracy. Prospective implementation studies are required to determine whether integrating such models into emergency department workflows improves triage decisions, resource allocation, or patient outcomes.

Finally, electronic health record-based automation and artificial intelligence-assisted monitoring may allow real-time calculation of physiological risk scores after CT-confirmed cervical spine fractures [21]. However, any automated decision-support tool should undergo external validation, calibration assessment, and clinical impact evaluation before its implementation.

## 5. Conclusions

In adult patients with CT-confirmed cervical spine fractures and major trauma severity, the MAP-derived Shock Index provided incremental prognostic information for in-hospital mortality beyond routinely available clinical variables and ISS in this retrospective cohort. These findings suggest that early physiological assessment may complement imaging-based, anatomical, and neurological evaluations; however, it should not replace these established components of cervical spine trauma assessment. Because the model was developed in a single-center cohort and underwent internal validation only, external multicenter validation is required before clinical implementation of the model.

## Figures and Tables

**Table 1:** Baseline demographic, injury, and emergency department characteristics of the ISS ≥ 15 cohort.

Variable	Total (*n* = 131)	Survivors (*n* = 101)	Non-Survivors (*n* = 30)
Age, years	56 (38–69)	54 (36–66)	68 (44–78)
Male sex, n (%)	98 (74.8)	77 (76.2)	21 (70.0)
Mechanism: Fall, *n* (%)	34 (26.0)	25 (24.8)	9 (30.0)
Mechanism: Motor vehicle-related, *n* (%)	93 (71.0)	72 (71.3)	21 (70.0)
Upper-cervical fracture, *n* (%)	52 (39.7)	39 (38.6)	13 (43.3)
Subaxial/other cervical fracture, *n* (%)	79 (60.3)	62 (61.4)	17 (56.7)
ED intubation, *n* (%)	38 (29.0)	17 (16.8)	21 (70.0)
Comorbidity, *n* (%)	77 (58.8)	52 (51.5)	25 (83.3)
ISS, median (IQR)	34 (26–43)	34 (25–41)	43 (35–57)
MAP-derived Shock Index	0.97 (0.83–1.17)	0.91 (0.82–1.04)	1.25 (1.07–1.79)

Abbreviations: ED, emergency department; ISS, Injury Severity Score; IQR, interquartile range. Motor vehicle-related mechanisms include in-vehicle, pedestrian, and motorcycle trauma. Values are presented as median (IQR) or *n* (%).

**Table 2:** Apparent and optimism-corrected performance of mortality prediction models.

Model	Apparent AUC (95% CI)	Optimism-Corrected AUC	Brier	Optimism-Corrected Brier	ΔAUC vs. Base	NRI vs. Base	IDI vs. Base
Base	0.86 (0.79–0.93)	0.84	0.12	0.14	—	—	—
Base + MAP-derived SI	0.93 (0.87–0.97)	0.91	0.09	0.10	+0.07	+0.98	+0.179
Base + ISS	0.87 (0.79–0.93)	0.84	0.12	0.13	+0.00	+0.25	+0.024
Base + MAP-derived SI + ISS	0.93 (0.87–0.97)	0.90	0.09	0.10	+0.06	+0.98	+0.180

Abbreviations: AUC, area under the receiver operating characteristic curve; SI, MAP-derived Shock Index; ISS, Injury Severity Score; NRI, continuous net reclassification improvement; IDI, integrated discrimination improvement. The NRI and IDI were reported relative to the base model.

## Data Availability

The datasets generated and/or analyzed during the current study are not publicly available because of institutional data protection regulations and ethical restrictions. De-identified data may be made available from the corresponding author upon reasonable request, subject to institutional approval.

## Supplementary Materials

The following supporting information can be downloaded at: https://www.mdpi.com/article/10.3390/diagnostics16142272/s1. Supplementary Figure S1: Flow diagram of patient selection; Supplementary Figure S2: Calibration plots for candidate models; Supplementary Table S1: Shrinkage-adjusted coefficients of the final prognostic model; Supplementary Table S2: Correlation and reclassification analyses; Supplementary Table S3: Sensitivity and exploratory model performance; Supplementary Table S4: Variable-level missingness; Supplementary File S1: Analysis code; Supplementary File S2: Excel-based bedside mortality risk calculator.
