# Supplementary material for: MAP-Derived Shock Index for Point-of-Care Physiological Risk Stratification After CT-Confirmed Cervical Spine Fracture: Development and Internal Validation of a Prognostic Model for In-Hospital Mortality
Source: Diagnostics (Basel). 2026 Jul 21;16(14):2272. doi: 10.3390/diagnostics16142272 (PMC13408033; doi:10.3390/diagnostics16142272)
Supplement: Supplementary file 1 [file diagnostics-16-02272-s001.zip › diagnostics-4380791-supplementary.pdf]

Supplementary Materials

MAP-Derived Shock Index for Point-of-Care Physiological Risk Stratification After CT-Confirmed Cervical Spine Fracture

Data source: Supplementary analyses were generated from the analytic dataset containing 131 eligible patients. The MAP-derived Shock Index was calculated as heart rate divided by estimated systolic pressure, where estimated systolic pressure = 1.5 x mean arterial pressure. This variable is not the conventional systolic blood pressure-based Shock Index and is provided as a MAP-derived approximation.

Supplementary Figure S1. Flow diagram of the analytic cohort

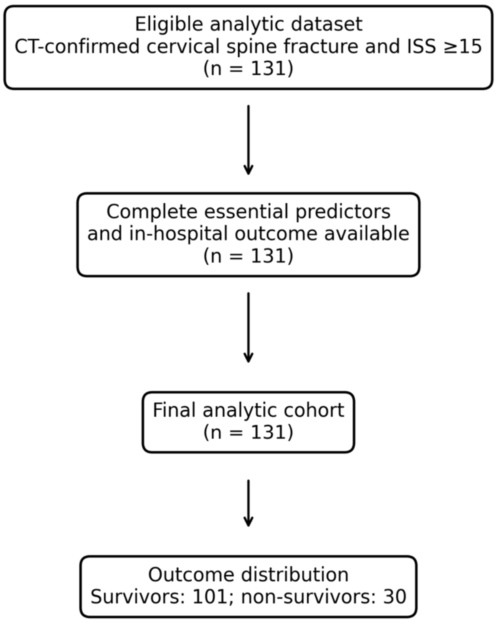

Supplementary Figure S1. Flow diagram of the analytic cohort used for model development and internal validation.

Supplementary Figure S2. Calibration plot of the final prognostic model

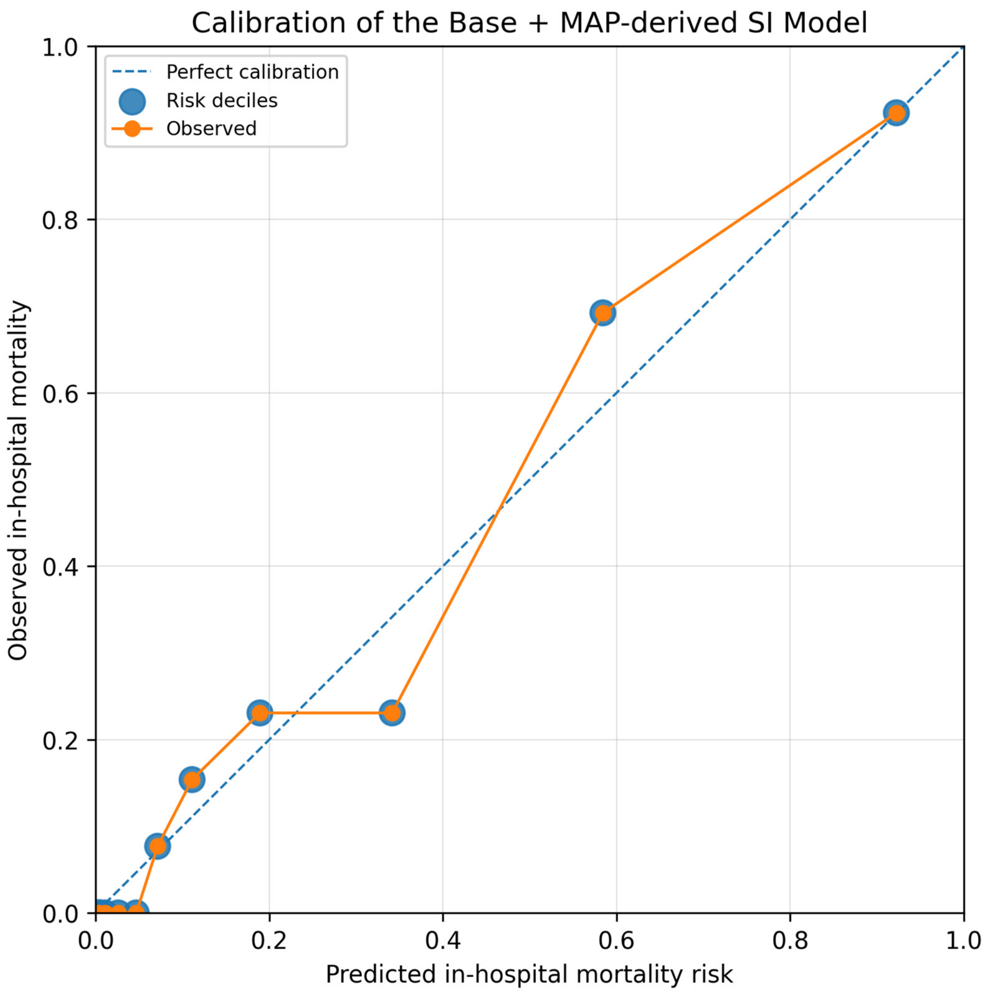

Supplementary Figure S2. Calibration of the Base + MAP-derived Shock Index model across predicted-risk deciles. Bubble size reflects the number of patients in each decile.

Supplementary Table S1. Final model coefficients and shrinkage-adjusted coefficients

Predictor	β	SE	OR	95% CI for OR	p value	Shrinkage-adjusted β	Shrinkage-adjusted OR
Intercept	-9.996	2.641	0.00	0.00–0.01	<0.001	-9.272	0.00
Age	0.062	0.022	1.06	1.02–1.11	0.005	0.057	1.06
GCS	-0.039	0.132	0.96	0.74–1.25	0.769	-0.036	0.96
Comorbidity	0.900	0.698	2.46	0.63–9.66	0.197	0.829	2.29
ED intubation	2.037	1.260	7.67	0.65–90.66	0.106	1.875	6.52
MAP-derived SI	3.617	0.983	37.24	5.42–255.88	<0.001	3.329	27.92

Notes: The final model was the Base + MAP-derived SI model. Uniform shrinkage was estimated using heuristic shrinkage based on model likelihood ratio chi-square: shrinkage factor = 0.920. The intercept was recalibrated to the observed mortality rate (22.9%). SI = MAP-derived Shock Index; ISS = Injury Severity Score; OR = odds ratio; SE = standard error. For clinical interpretability, the MAP-derived Shock Index effect can also be expressed per 0.1-unit increase: OR 1.72 (95% CI 1.29-2.30).

Supplementary Table S2. Correlation and reclassification analyses

Analysis	Comparison	Estimate	95% CI	p value
Spearman correlation	MAP-derived SI vs ISS	0.49	—	<0.001
Continuous NRI	Base + MAP-derived SI vs Base + ISS	1.04	0.68–1.41	—
IDI	Base + MAP-derived SI vs Base + ISS	0.155	0.076–0.248	—
Continuous NRI	Base + MAP-derived SI + ISS vs Base + MAP-derived SI	0.06	-0.33–0.48	—
IDI	Base + MAP-derived SI + ISS vs Base + MAP-derived SI	0.001	-0.001–0.002	—

Notes: Continuous NRI and IDI were calculated using apparent predicted probabilities from the fitted logistic models. Confidence intervals were estimated by nonparametric bootstrap resampling of patients. NRI = net reclassification improvement; IDI = integrated discrimination improvement.

Supplementary Table S3. Apparent performance of candidate and exploratory models

Model	AUC	95% CI for AUC	Brier score	95% CI for Brier score
Base	0.862	0.793–0.924	0.122	0.086–0.163
Base + MAP-derived SI	0.928	0.879–0.969	0.086	0.054–0.121
Base + ISS	0.865	0.793–0.927	0.118	0.081–0.154
Base + MAP-derived SI + ISS	0.927	0.873–0.968	0.085	0.055–0.118
Base + Lactate	0.870	0.793–0.933	0.115	0.077–0.153
Base + Base deficit	0.881	0.815–0.940	0.113	0.079–0.148
MAP-derived SI alone	0.819	0.713–0.908	0.131	0.092–0.172
ISS alone	0.742	0.632–0.838	0.150	0.113–0.189

Notes: Confidence intervals were estimated by nonparametric bootstrap resampling of apparent predicted probabilities. The exploratory laboratory-augmented models included lactate or base deficit added separately to the base clinical model.

Supplementary Table S4. Variable-level missingness in the provided analytic dataset

Variable	Total n	Missing n	Missing %
Age	131	0	0.0
Sex	131	0	0.0
Mechanism of injury	131	0	0.0
ISS	131	0	0.0
GCS	131	0	0.0
Pressure variable recorded as MAP	131	0	0.0
Heart rate	131	0	0.0
MAP-derived SI	131	0	0.0
Respiratory rate	131	0	0.0
Temperature	131	0	0.0
Comorbidity	131	0	0.0
ED intubation	131	0	0.0
Lactate	131	0	0.0
Base deficit	131	0	0.0
In-hospital mortality	131	0	0.0

Notes: Missingness was calculated from the uploaded spreadsheet used for the present supplementary analysis. No missing values were detected for the listed primary model variables in the provided analytic dataset.
